# pH-Self-Buffering and Flocculation-Enabled Nonradical Oxidation via Magnesium Hydroxide-Activated Peroxymonosulfate for Selective Organic Pollutant Degradation

**DOI:** 10.3390/nano16030166

**Published:** 2026-01-26

**Authors:** Yunfeng Zhang, Cheng Zhao, Zhongqun Li, Dexin Kong, Lingshuai Kong

**Affiliations:** 1 Shandong Provincial Geo-Mineral Engineering Exploration Institute, Shandong Provincial Bureau of Geology & Mineral Resources, Jinan 250014, China; 2 Shandong Engineering Research Center for Environmental Protection and Remediation on Groundwater, Jinan 250014, China; 3 Key Laboratory of Geological Disaster Risk Prevention and Control, Emergency Management Department of Shandong Province, Jinan 250014, China; 4 Institute of Eco-Environmental Forensics, School of Environmental Science and Engineering, Shandong University, Qingdao 266237, China; 202212150@mail.sdu.edu.cn

**Keywords:** magnesium hydroxide, peroxymonosulfate, electron-rich pollutants, pH self-regulation, singlet oxygen

## Abstract

Peroxymonosulfate (PMS)-based advanced oxidation is often hindered by pH instability and the lack of post-reaction separation. Herein, commercial magnesium hydroxide (Mg(OH)_2_) is introduced as a multifunctional catalyst to address these limitations. Mg(OH)_2_ effectively catalyzed PMS decomposition via a nonradical pathway dominated by singlet oxygen (^1^O_2_) generation, achieving rapid and complete degradation of electron-rich pollutants like bisphenol A (BPA) within 40 min. The system exhibits exceptional pH self-regulation, stabilizing the solution at ~9.8 and maintaining high efficiency across an initial pH range of 3–11. Mechanistic studies confirm ^1^O_2_ as the primary reactive species with a steady-state concentration of 1.67 × 10^−12^ M. The catalyst demonstrates strong resistance to common anions and humic acid, along with excellent stability over four cycles. Furthermore, Mg(OH)_2_ enables in situ flocculation and removal of degradation products. This work highlights Mg(OH)_2_ as an efficient, stable, and multifunctional activator, offering a integrated strategy for practical wastewater treatment.

## 1. Introduction

Clean water is essential for human health and development, often requiring the removal of contaminants from surface water or wastewater [1]. Refractory, low-concentration, and non-biodegradable organic compounds represent a significant class of environmental pollutants [2]. In recent years, peroxymonosulfate (PMS, HSO_5_^−^)-based advanced oxidation processes (AOPs) have garnered considerable attention for wastewater treatment due to their strong oxidative capacity [3,4]. These systems effectively degrade diverse organic pollutants, including emerging contaminants and recalcitrant waste streams [5]. The oxidation power primarily stems from reactive species generated upon PMS activation, such as sulfate radicals (SO_4_^•−^), hydroxyl radicals (•OH), singlet oxygen (^1^O_2_), and/or high-valent metals [6,7]. Catalysts, particularly transition metals or carbon-based materials, are typically employed to cleave the peroxide bond in PMS, initiating these reactions [8,9]. The activation mechanism, which proceeds via radical or non-radical pathways (e.g., involving ^1^O_2_, high-valent metal-oxo species, or direct electron transfer), critically determines the selectivity and efficiency of the degradation process [10,11,12,13].

To achieve selective pollutant degradation and enhance practical applicability, recent studies have focused on designing heterogeneous catalysts that direct PMS activation toward specific non-radical pathways. For example, Co_3_O_4_/carbon nanotubes, biochar-supported vanadiferous magnetite composites, and nitrogen-doped carbon materials have demonstrated the ability to selectively degrade electron-rich organic pollutants via a ^1^O_2_-dominated pathway, which offers improved resistance to background matrix interference [14,15,16]. Furthermore, catalysts engineered with distinct surface properties, such as beneficial metal cations, heteroatom doping, oxygen vacancies, or basic sites, have been shown to promote direct electron transfer or the formation of high-valent metal-oxo species, thereby also contributing to selective oxidation processes [17,18,19,20,21]. Despite these advances, many such sophisticated catalyst systems still face challenges including potential metal leaching, a limited operational pH range, and the lack of inherent functionality for separating reaction intermediates or the spent catalyst after use. Therefore, developing robust and multifunctional heterogeneous catalysts that integrate efficient and selective PMS activation with broad pH adaptability and facile separation remains a significant challenge.

A key operational constraint is the inherent acidification of the solution due to H^+^ release from PMS decomposition, which can impair oxidative efficiency and system stability [22,23]. While various catalysts have been explored to enhance PMS activity across different pH ranges [24,25,26], most systems lack integrated functionality for post-treatment separation. Unlike conventional Fenton processes where Fe^3+^ hydrolysis facilitates flocculation of degradation products [27], PMS-based AOPs often operate under mildly acidic conditions (around pH 5), hindering the in situ formation of effective flocculants and complicating the removal of reaction intermediates.

Magnesium hydroxide (Mg(OH)_2_), a naturally occurring alkaline mineral (brucite), features a layered crystal structure composed of Mg^2+^ ions coordinated with hydroxyl groups [28]. As an engineered nanomaterial, it is widely used in flame retardancy, pharmaceuticals, and materials science owing to its moderate alkalinity, low solubility, stability, and cost-effectiveness [29]. In environmental applications, Mg(OH)_2_ is commonly employed for pH adjustment, neutralization, and contaminant removal via precipitation and flocculation, leveraging its surface adsorption and charge-neutralization capabilities [30,31,32,33]. Preliminary evidence suggests that surface hydroxyl groups on MgO can activate PMS to generate ^1^O_2_, enabling selective degradation of electron-rich organics [34]. We therefore hypothesize that Mg(OH)_2_ can serve as a multifunctional persulfate activator, capable of simultaneously catalyzing PMS decomposition, buffering the reaction pH, and enhancing the flocculation and removal of degradation products—thus addressing critical gaps in conventional PMS-AOPs.

Herein, we develop and evaluate a novel Mg(OH)_2_-activated PMS system. This work aims to utilize the inherent alkalinity of Mg(OH)_2_ to neutralize reaction-induced acidity, thereby stabilizing pH and maintaining high oxidation efficiency. Concurrently, we exploit its flocculation properties to enable the efficient separation and removal of degraded organic products. This innovative catalyst not only improves the oxidation efficiency of PMS but also enables more efficient and environmentally friendly pollutant removal, making it a promising solution for advanced wastewater treatment.

## 2. Materials and Methods

### 2.1. Chemicals

Magnesium hydroxide (Mg(OH)_2_, ~100 nm), peroxymonosulfate (PMS, 2KHSO_5_·KHSO_4_·K_2_SO_4_, ≥47.0% KHSO_5_ basis), 5,5-dimethyl-1-pyrroline N-oxide (DMPO), 4-amino-2,2,6,6-tetramethylpiperidine (TEMP), bisphenol A (C_15_H_16_O_2_, BPA), humic acid (HA), p-benzoquinone (BQ), and furfuryl alcohol (FFA) were purchased from Aladdin Chemical Co., Ltd. (Shanghai, China). 4-chlorophenol (4-CP), phenol (BP), rhodamine 6G (Rh6G), tert-butyl alcohol (TBA), and nitrobenzene (NB) were obtained from Sinopharm Chemical Reagent Co., Ltd. (Shanghai, China). Methanol and ethanol were obtained from Tianjin Fu Yu Fine Chemicals Co., Ltd. (Tianjin, China). All chemicals were of analytical grade and used as received. All solutions were prepared using deionized water (18.25 MΩ·cm).

### 2.2. Catalytic Degradation Experiments

All batch experiments were conducted in the dark at 25 ± 2 °C with magnetic stirring (EMS-40, Shanghai Joyn Electronic Co., Ltd. Shanghai, China). In a typical procedure, 25 mg of Mg(OH)_2_ catalyst was dispersed into 35 mL of deionized water in a 100 mL conical flask and sonicated for 1 min. Subsequently, 10 mL of a BPA stock solution (25 mg L^−1^) was added to achieve a final concentration of 22 µM (50 mL total volume). The initial pH was adjusted using 0.1 M NaOH or H_2_SO_4_. The suspension was stirred in the dark for 20 min to establish adsorption–desorption equilibrium before initiating the reaction by adding 5 mL of a PMS solution (final concentration: 0.6 mM). At predetermined time intervals, 2 mL aliquots were withdrawn and immediately quenched with 0.5 mL of furfuryl alcohol, then filtered through a 0.22 µm membrane for analysis. Experiments for other pollutants (NB, 4-CP, BP, Rh6G, each at 22 µM) and comparative catalysts (NaOH, Al(OH)_3_, and Ca(OH)_2_) followed the same protocol. All experiments were performed in triplicate. The degradation efficiency (*DE*, %) of pollutants were calculated using Equation (1):

(1) $$DE\;\left(\%\right)=\frac{\left(C_{0}-C_{t}\right)}{C_{0}}\times 100\%$$

where *C*_0_ represents the initial concentration of pollutant (mg/L) and *C_t_* represents its concentration at reaction time *t*. The value of *C_t_*/*C*_0_ represents the ratio of pollutant concentration at time t to its initial concentration (0 min). For reusability tests, the spent catalyst was recovered after each cycle via centrifugation, washed thoroughly with ethanol and deionized water, dried at 60 °C under vacuum, and then reused under identical conditions.

### 2.3. Analytical Methods and Characterization

The concentration of BPA was quantified by high-performance liquid chromatography (HPLC, ELITE P2201) equipped with a C18 column, using a methanol/water (70:30, *v*/*v*) mobile phase at a flow rate of 1.0 mL min^−1^ and a detection wavelength of 278 nm. The method detection limit for BPA was 0.05 mg L^−1^. Total organic carbon was measured using a Shimadzu TOC-V CPN analyzer. Residual PMS concentration was determined by a spectrophotometric method at the maximum absorbance peak of 350 nm [35]. Reactive oxygen species were identified by electron paramagnetic resonance (EPR) spectroscopy (JES–X320) using DMPO and TEMP as spin-trapping agents. The crystal structure of the catalyst was characterized by powder X-ray diffraction (XRD, Bruker D8) with Cu K_α_ radiation (*λ* = 0.1542 nm). The sizes and morphologies of the samples were characterized by field-emission scanning electron microscopy (FE-SEM, Zeiss Gemini 300, Oberkochen, Germany). Surface elemental composition and chemical states were analyzed by X-ray photoelectron spectroscopy (XPS, Thermo ESCALAB 250XI, Waltham, MA, USA), with binding energies calibrated against the C 1s peak at 284.8 eV. Fourier transform infrared (FT-IR) spectra of the catalysts were obtained using a Bruker VERTEX-70 spectrometer (Billerica, MA, USA). The measurements were performed in the range of 4000 to 400 cm^−1^ with samples prepared as potassium bromide (KBr) pellets. 

## 3. Results and Discussion

### 3.1. Characterization of Mg(OH)_2_

**Figure 1:**
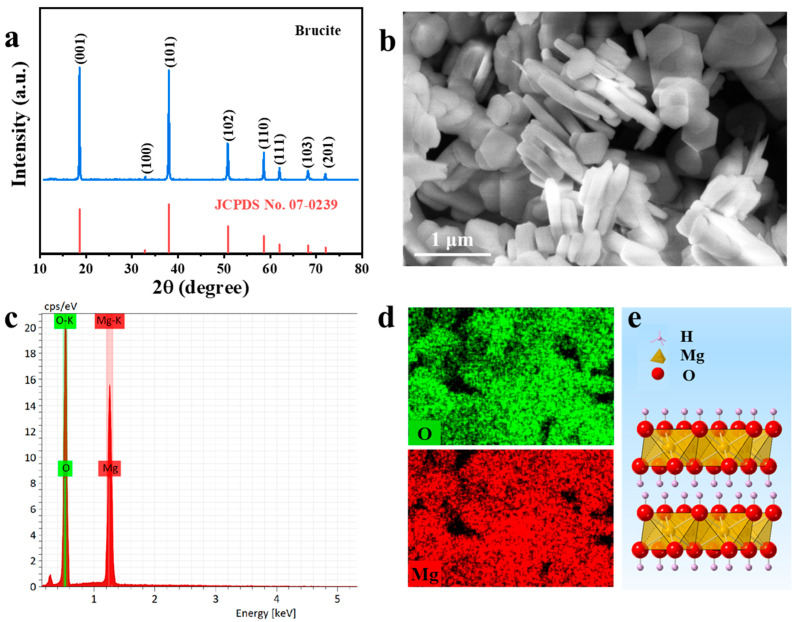
Characterizations of Mg(OH)_2_: (**a**) XRD pattern, (**b**) FE-SEM image, (**c**) EDS spectra, (**d**) EDS mapping images. (**e**) Schematic diagram of Mg(OH)_2_ crystal structure.

The X-ray diffraction (XRD) pattern of the commercial Mg(OH)_2_ is presented in Figure 1a. All diffraction peaks can be indexed to the hexagonal brucite structure (space group *P-3 m1* (164), JCPDS No. 07-0239), with characteristic reflections at 18.6°, 32.8°, 38.0°, 50.8°, 58.6°, 62.1°, 68.2°, and 72.0°. The calculated lattice parameters were a = b = 3.147 Å and c = 4.769 Å. The sharp diffraction peaks indicate high crystallinity, which was quantitatively estimated to be ~98.74%. No impurity phases were detected, confirming the high phase purity of the Mg(OH)_2_ sample. The morphology of the Mg(OH)_2_, characterized by FE-SEM (Figure 1b), shows nanosheets approximately 90 nm thick and 0.6 µm in lateral size. Its elemental composition was determined by EDS, yielding an atomic ratio of 58.18% O and 41.82% Mg (Figure 1c). This composition is corroborated by the corresponding elemental mapping, which confirms the homogeneous distribution of O and Mg across the nanosheets (Figure 1d). Schematic diagram of Mg(OH)_2_ crystal structure was show in Figure 1e.

**Figure 2:**
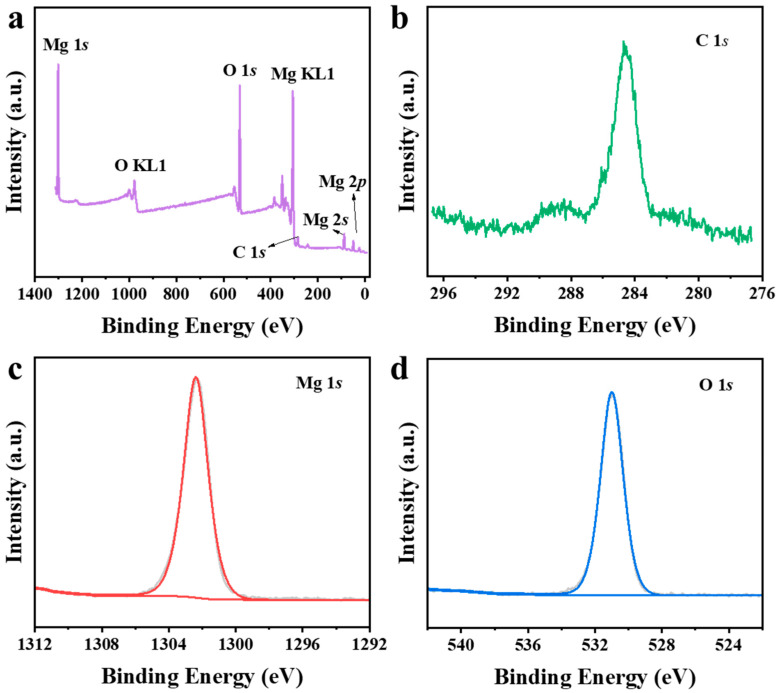
High-resolution XPS spectra of (**a**) survey, (**b**) C 1s, (**c**) Mg 1s, and (**d**) O 1s.

The surface chemical composition was analyzed by X-ray photoelectron spectroscopy (XPS) (Figure 2). The survey spectrum (Figure 2a) shows predominant signals of Mg, O, and C. The C 1s peak at 284.8 eV was used as a charge reference (Figure 2b) [36,37]. The high-resolution Mg 1s spectrum (Figure 2c) exhibits a peak at 1302.4 eV, corresponding to Mg^2+^ in Mg(OH)_2_ [38,39]. The O 1s spectrum (Figure 2d) shows a main component at 531.0 eV, attributable to oxygen in hydroxyl groups (–OH) [40]. These results collectively indicate that the surface of the material is predominantly composed of Mg(OH)_2_.

### 3.2. Catalytic Performance Evaluation

**Figure 3:**
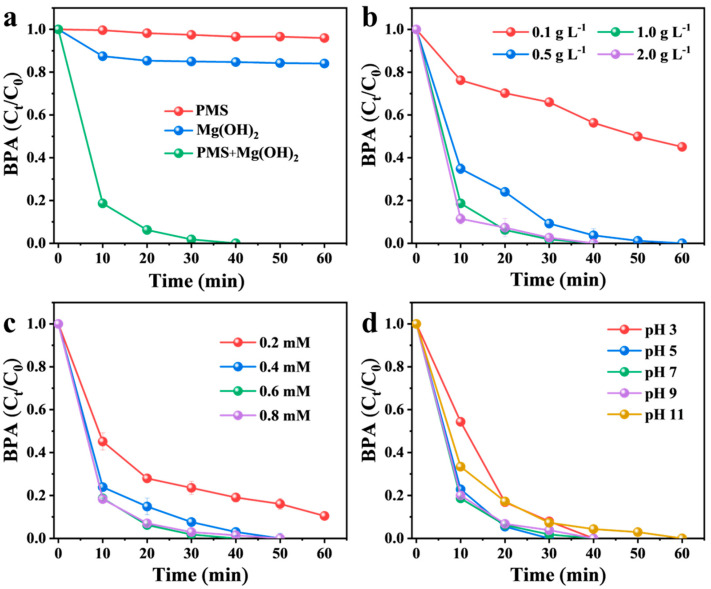
(**a**) Catalytic performance of Mg(OH)_2_ in activating PMS for BPA degradation. Effects of (**b**) Mg(OH)_2_ loading, (**c**) PMS concentration, and (**d**) initial pH on BPA degradation. Reaction conditions: (**a**) [Mg(OH)_2_]_0_ = 1 g L ^− 1^; [PMS]_0_ = 0.6 mM; [BPA]_0_ = 22 µM; initial pH 7. (**b**) [Mg(OH)_2_]_0_ = 0.1–2.0 g L^−1^; [PMS]_0_ = 0.6 mM; [BPA]_0_ = 22 µM; initial pH 7. (**c**) [Mg(OH)_2_]_0_ = 1 g L^−1^; [PMS]_0_ = 0.2–0.8 mM; [BPA]_0_ = 22 µM; initial pH 7. (**d**) [Mg(OH)_2_]_0_ = 1 g L^−1^; [PMS]_0_ = 0.6 mM; [BPA]_0_ = 22 µM; initial pH 3–11.

Figure 3a shows that approximately 16% of BPA was adsorbed onto the Mg(OH)_2_ surface, which may be attributed to the adsorption and flocculation capabilities of Mg(OH)_2_. In contrast, PMS alone (without catalyst) degraded only about 4% of BPA over 60 min, suggesting its intrinsic oxidizing capacity was negligible. However, in the Mg(OH)_2_/PMS system, BPA was rapidly degraded, with 82% removal within 10 min and complete elimination achieved within 40 min. Furthermore, a comparative analysis of catalytic performance for PMS activation (Table S1) demonstrates that Mg(OH)_2_ exhibits competitive, and in some cases enhanced, performance relative to other high-performance catalysts documented for ^1^O_2_-mediated BPA degradation.

The effect of catalyst loading is presented in Figure 3b. The dosage of Mg(OH)_2_ significantly influenced the degradation efficiency of BPA. The degradation performance improved markedly with increasing catalyst loading. Specifically, a low loading of 0.1 g/L achieved only 55% BPA removal after 60 min, while 0.5 g/L enabled complete (100%) degradation within the same timeframe. Further increasing the loading to 1.0 and 2.0 g/L accelerated the process, achieving complete degradation within 50 and 40 min, respectively. This clear positive correlation between the Mg(OH)_2_ amount and the degradation efficiency indicates that the reaction kinetics were primarily governed by the availability of surface active sites. A higher catalyst dosage provides a greater number of active sites (e.g., surface hydroxyl groups) for activating PMS, thereby enhancing the generation of reactive species and leading to faster and more complete pollutant removal.

As depicted in Figure 3c, PMS concentration also significantly influenced degradation performance. At PMS concentrations below 0.2 mM, complete BPA degradation required more than 60 min. Increasing PMS concentration to 0.4 mM reduced the time required for 100% BPA removal to 50 min, which can be attributed to enhanced generation of reactive species, thereby increasing the probability of attack on BPA molecules and accelerating degradation kinetics [41]. Further increasing PMS concentration to 0.6 mM and 0.8 mM continued to improve degradation rates; however, no further kinetic enhancement was observed at 0.8 mM compared to 0.6 mM, likely due to saturation of reactive species or scavenging effects at elevated oxidant levels [42].

Solution pH is a critical parameter affecting both catalyst speciation and PMS activation. Figure 3d presents the BPA degradation profiles in the Mg(OH)_2_/PMS under different initial pH conditions. Notably, complete BPA removal was achieved within 60 min across a broad initial pH range of 3–11. Although solution pH typically governs PMS activation, the degradation efficiency of the Mg(OH)_2_/PMS system remained largely unaffected by varying the initial pH from 3 to 11. This pH-independent performance is attributed to the buffering effect of Mg(OH)_2_, which stabilized the solution pH at approximately 9.8 upon its addition regardless of the initial acid value [43]. It is noteworthy that complete BPA degradation was sustained even under alkaline conditions, demonstrating remarkable insensitivity to pH variation. Such pH-independent behavior, commonly associated with nonradical oxidation pathways, represents a distinct advantage of the Mg(OH)_2_/PMS system over many conventional catalysts that exhibit strong pH-dependent activity. Based on these results, a catalyst dosage of 1 g/L and a PMS concentration of 0.6 mM were selected for all subsequent experiments.

### 3.3. Possible Activation Mechanism

**Figure 4:**
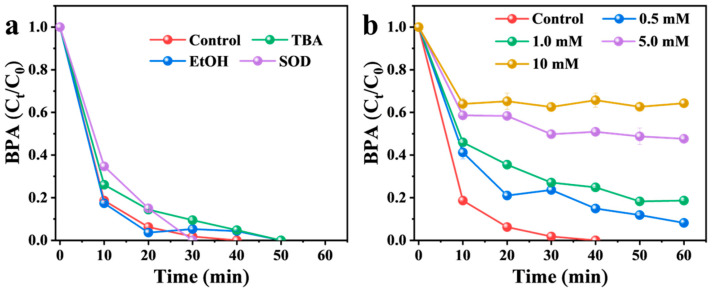
Effects of different quenchers on BPA degradation in the Mg(OH)_2_/PMS system: (**a**) EtOH, TBA, SOD, and (**b**) FFA.

Quenching experiments were conducted to identify the reactive species generated in the Mg(OH)_2_/PMS system. Ethanol (EtOH) and tert-butyl alcohol (TBA) were employed as scavengers to probe the potential roles of •OH and SO_4_^•−^, respectively [44,45]. As shown in Figure 4a, the degradation of BPA was not significantly suppressed upon the addition of excess EtOH or TBA, suggesting limited contributions from these radical species. To examine the involvement of superoxide radical (O_2_^•−^), superoxide dismutase (SOD, 500 U mL^−1^) was introduced as a selective quencher [46]. The degradation efficiency of BPA remained nearly unchanged, indicating that O_2_^•−^ did not play a major role in the reaction. Furthermore, furfuryl alcohol (FFA, 0.5–10 mM) was used as a selective scavenger to evaluate the non-radical pathway involving singlet oxygen (^1^O_2_), based on its high and specific reaction rate with ^1^O_2_ (k = 1.2 × 10^8^ M^−1^ s^−1^) as reported in the literature [12,47]. As depicted in Figure 4b, the addition of FFA markedly inhibited BPA degradation in a concentration-dependent manner. When the FFA concentration increased from 0.5 to 10 mM, the degradation efficiency was progressively suppressed, with 10 mM FFA reducing it from 100% to 35% within 40 min. This result confirms that ^1^O_2_ serves as a crucial non-radical reactive species responsible for BPA degradation in the Mg(OH)_2_/PMS system.

**Figure 5:**
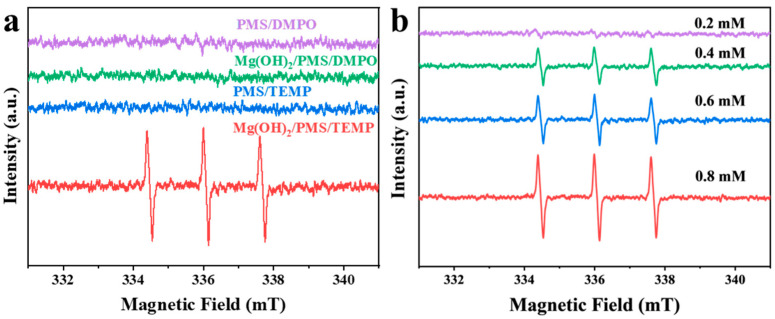
(**a**) EPR spectra recorded in the presence of DMPO or TEMP under different conditions: Mg(OH)_2_/PMS system and PMS alone. (**b**) EPR spectra for the detection of ^1^O_2_ (using TEMP as the spin-trapping agent) in the Mg(OH)_2_/PMS system at varying PMS concentrations.

To elucidate the active species involved in the Mg(OH)_2_/PMS system, electron paramagnetic resonance (EPR) spectroscopy was carried out using 5,5-dimethyl-1-pyrroline N-oxide (DMPO) and 2,2,6,6-tetramethylpiperidine (TEMP) as spin-trapping agents [48]. DMPO was first employed to probe the generation of radical species, including •OH, SO_4_^•−^, and O_2_^•−^. As shown in Figure 5a, no characteristic signals were detected in the Mg(OH)_2_/PMS/DMPO system, indicating that these radicals were not produced—a finding consistent with the results of the quenching experiments. When TEMP was used as the trapping reagent for ^1^O_2_, a distinct triplet signal with intensity corresponding to that of TEMP–^1^O_2_ adduct was observed (Figure 5b). The signal intensity increased with higher PMS dosage, suggesting that more PMS participated in the catalytic reaction to generate ^1^O_2_, thereby enhancing pollutant degradation. This trend aligns well with the degradation performance shown in Figure 3c.

**Figure 6:**
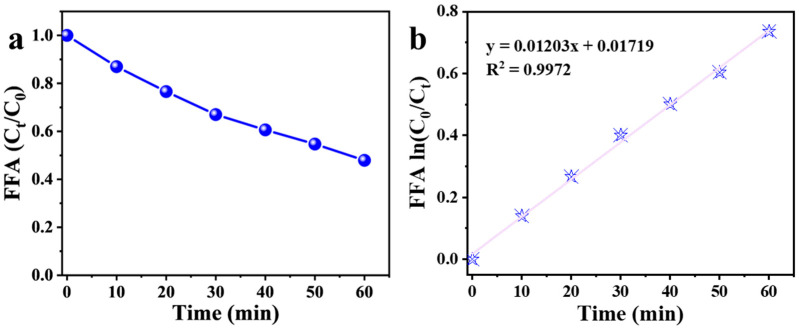
(**a**) Determination of [^1^O_2_]_SS_ from the kinetic of furfuryl alcohol (FFA) degradation in Mg(OH)_2_/PMS system. (**b**) pseudo-first-order fitting of FFA concentration decay in Mg(OH)_2_/PMS system.

To further quantify the steady-state concentration of ^1^O_2_ ([^1^O_2_]_SS_), FFA was employed as a selective probe following established methods (Figure 6a) [49,50]. The decay of FFA followed pseudo-first-order kinetics, as evidenced by the linear relationship between ln[C_0_]/[C_t_] and reaction time under steady-state ^1^O_2_ conditions (Figure 6b). The slope of this linear plot was divided by the second-order rate constant for the reaction between ^1^O_2_ and FFA (1.2 × 10^8^ M^−1^ s^−1^) to determine [^1^O_2_]_SS_. Based on the kinetic data obtained in the absence of FFA, the calculated steady-state concentration of ^1^O_2_ was 1.67 × 10^−12^ M.

**Figure 7:**
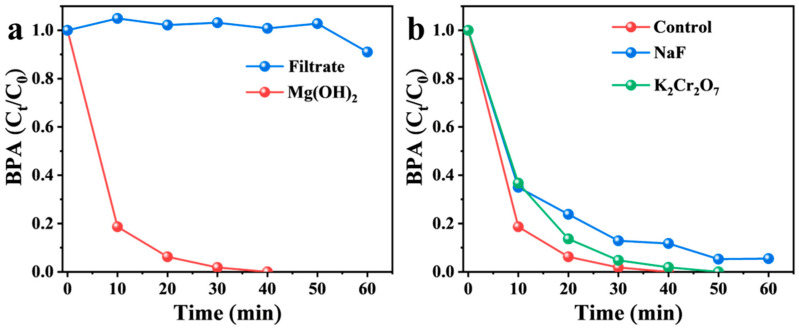
(**a**) Catalytic performance of Mg(OH)_2_ filtrate in activating PMS for BPA degradation. (**b**) Effects of NaF and K_2_Cr_2_O_7_ on BPA degradation in Mg(OH)_2_/PMS system. Reaction conditions: [Mg(OH)_2_]_0_ = 1 g L^−1^; [PMS]_0_ = 0.6 mM; [BPA]_0_ = 22 µM; [NaF]_0_ = 5 mM; [K_2_Cr_2_O_7_]_0_ = 1 mM; “Control” refers specifically to the Mg(OH)_2_/PMS/BPA system without the addition of any scavengers (i.e., neither NaF nor K_2_Cr_2_O_7_).

As shown in Figure 7a, negligible BPA degradation was observed in the filtrate of the Mg(OH)_2_ suspension, indicating that the solid Mg(OH)_2_ phase, rather than leached magnesium ions or homogeneous OH^−^, played the dominant catalytic role. In PMS activation systems, direct electron transfer and ^1^O_2_ oxidation are two predominant non-radical pathways for pollutant degradation. To probe the potential contribution of electron transfer, potassium dichromate (K_2_Cr_2_O_7_) was employed as an effective quencher [51]. The degradation efficiency of BPA showed no significant decrease upon the addition of K_2_Cr_2_O_7_ (Figure 7b), suggesting that an electron-transfer process did not govern the reaction in the Mg(OH)_2_/PMS system. Collectively, these results demonstrate that ^1^O_2_ acts as the primary reactive species responsible for BPA degradation.

To elucidate the formation mechanism of ^1^O_2_, the influence of sodium fluoride (NaF) on the degradation process was evaluated. Based on previous reports, ^1^O_2_ generation in such systems is often attributed to surface hydroxyl groups (≡OH), which can form hydrogen bonds with the –OH moiety of HSO_5_^−^ [52,53]. The interaction between two adjacent HSO_5_^−^ molecules leads to the formation of a peroxydisulfate-like intermediate, (SO_4_O)_2_^2−^, which subsequently decomposes rapidly into ^1^O_2_ and sulfate ions (Equations (2) and (3)) [34]. To verify whether this pathway operates in the present system, NaF was introduced. Fluoride ions (F^−^) are known to strongly interact with surface −OH groups via hydrogen bonding (e.g., −OH_ads_⋯F^−^), thereby displacing or passivating these active sites [54,55]. As shown in Figure 7b, the addition of F^−^ significantly suppressed BPA degradation. This result strongly supports the conclusion that surface hydroxyl groups on Mg(OH)_2_ are essential for activating PMS to generate ^1^O_2_, confirming the proposed mechanism.

(2) $$2{HSO}_{5}^{-}+2\equiv OH\to {\left({SO}_{4}O\right)}_{2}^{2-}+2H_{2}O$$

(3) SO4O22−→2SO42−+O21

**Figure 8:**
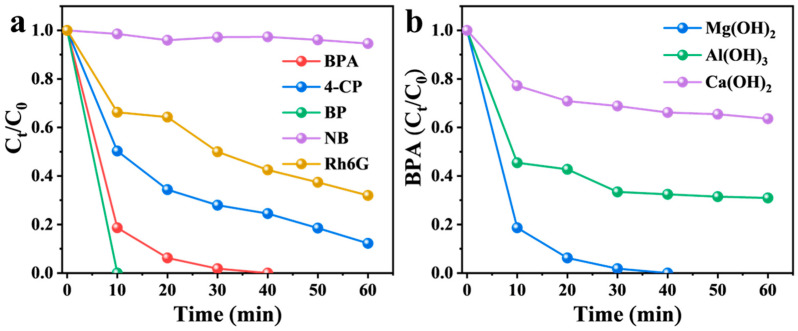
(**a**) Catalytic performance of Mg(OH)_2_ in the degradation of various pollutants. (**b**) kinetics of BPA degradation by PMS activated with other metal hydroxide activators. Reaction conditions: (**a**) [Mg(OH)_2_]_0_ = 1 g L^−1^; [PMS]_0_ = 0.6 mM; [pollutants]_0_ = 22 µM. (**b**) [catalyst]_0_ = 1 g L^−1^; [PMS]_0_ = 0.6 mM; [BPA]_0_ = 22 µM.

As shown in Figure 8a, phenol (BP) was completely degraded within 10 min in the Mg(OH)_2_/PMS system, whereas the degradation efficiencies for 4-chlorophenol (4-CP) and rhodamine 6G (Rh6G) reached 88% and 68%, respectively, after 60 min. In contrast, nitrobenzene (NB) was barely degraded under the same conditions. These results indicate that the Mg(OH)_2_/PMS system is effective for degrading various electron-rich phenolic contaminants, while exhibiting limited activity toward electron-deficient compounds. Furthermore, Al(OH)_3_ and Ca(OH)_2_, which share a similar crystalline structure with Mg(OH)_2_, were evaluated as catalysts under identical conditions. As shown in Figure 8b, the BPA degradation efficiencies obtained with Al(OH)_3_ and Ca(OH)_2_ were 69% and 36%, respectively, demonstrating that the catalytic performance is strongly dependent on the metal center, likely due to differences in surface hydroxyl density.

### 3.4. Evaluation of Flocculation Performance

**Figure 9:**
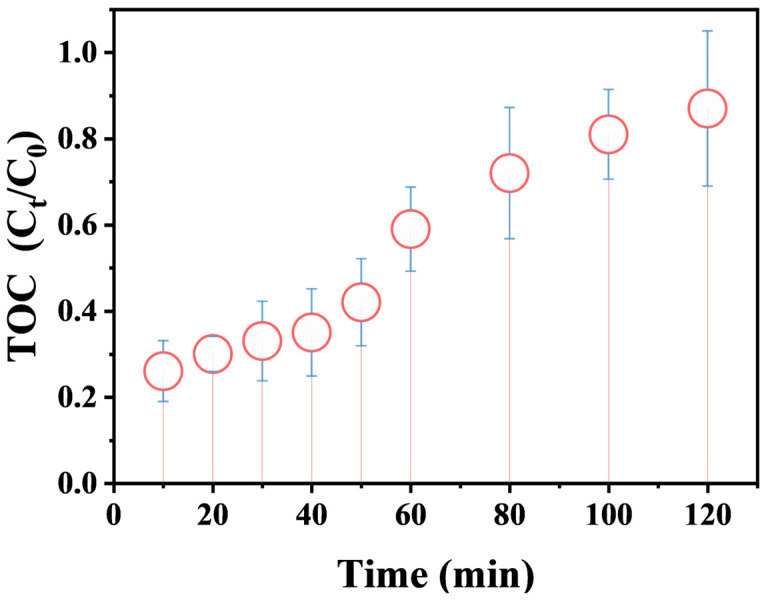
TOC removal efficiency of Mg(OH)_2_/PMS system in BPA degradation.

**Figure 10:**
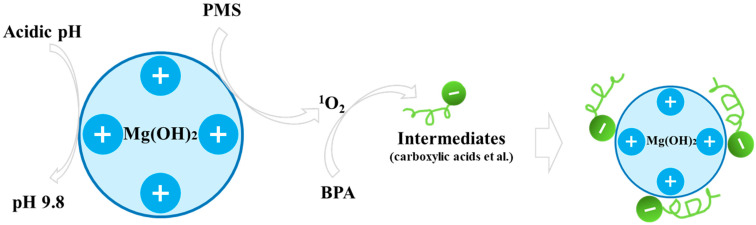
Reaction scheme for BPA degradation and flocculation in the Mg(OH)_2_/PMS system [56,57,58].

To quantitatively assess the flocculation performance, the variation in total organic carbon (TOC) of the supernatant was monitored during the reaction process (Figure 9). Although BPA was completely degraded within 40 min, the TOC removal at this point was only ~35%. As the reaction time was extended to 120 min, the TOC removal rate steadily increased to ~87%. This confirms that the flocculation primarily targets the generated intermediates rather than the parent BPA. The proposed flocculation mechanism involves the oxidation of BPA to hydrophilic acidic intermediates (e.g., acetic, oxalic, formic, propanedioic, or succinic acids) [56,57,58] by Mg(OH)_2_-activated PMS (primarily via ^1^O_2_), their subsequent dissociation into anions (e.g., R-COO^−^) under the buffered alkaline conditions, adsorption onto positively charged Mg(OH)_2_ colloids and Mg^2+^ via electrostatic/coordinative interactions, and final removal through sweep flocculation and sedimentation (Figure 10) [32]. Thus, Mg(OH)_2_ acts as a dual-functional catalyst-flocculant, enabling an integrated oxidation-flocculation strategy that enhances overall decontamination and simplifies solid–liquid separation.

### 3.5. Effect of Common Matrix Species

**Figure 11:**
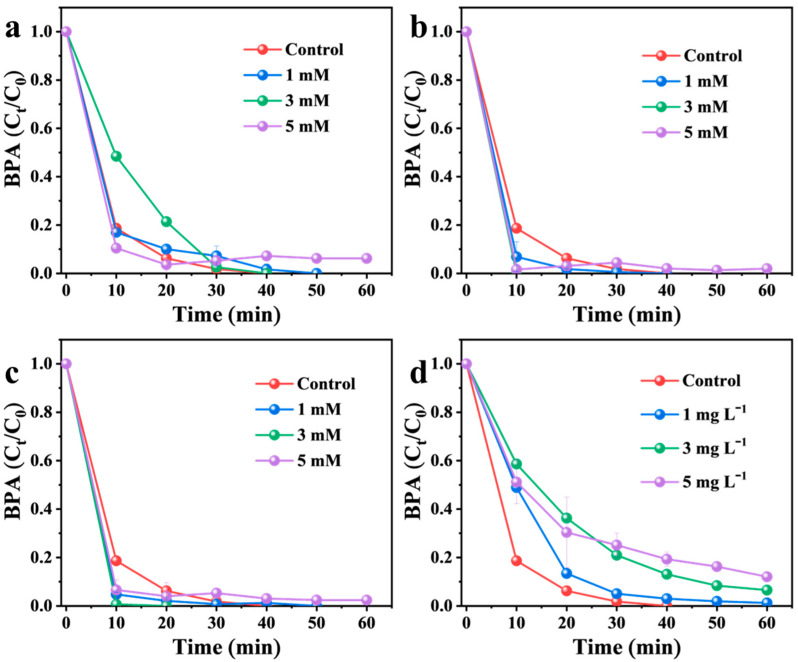
Effect of (**a**) Cl^−^, (**b**) HCO_3_^−^, (**c**) CO_3_^2−^, and (**d**) HA on the BPA degradation. Reaction conditions: [Mg(OH)_2_]_0_ = 1 g L^−1^; [PMS]_0_ = 0.6 mM; [BPA]_0_ = 22 µM; initial pH 7.

To evaluate the practical applicability of the present catalyst, the effects of common water matrix components, humic acid (HA) and inorganic anions (such as Cl^−^, CO_3_^2−^, and HCO_3_^−^) on BPA degradation were investigated under the standard reaction conditions (pH ~9.8, after added Mg(OH)_2_) (Figure 11). Inorganic anions, which are ubiquitous in natural waters, often significantly influence reactive oxygen species and thus pollutant removal. As shown in Figure 11a, increasing the Cl^−^ concentration from 1 to 5 mM had almost no effect on BPA degradation in the Mg(OH)_2_/PMS system. Similarly, the presence of 1–5 mM CO_3_^2−^ or HCO_3_^−^ did not cause significant inhibition of BPA removal (Figure 11b,c). It should be noted that at the system pH of ~9.8, the dominant inorganic carbon species is bicarbonate (HCO_3_^−^), with a minor fraction as carbonate (CO_3_^2−^). The added salts equilibrate accordingly within the buffered medium. These results highlight a distinct advantage of the ^1^O_2_-dominated non-radical pathway: its strong resistance to interference from common inorganic anions in real water matrices.

Humic acid (HA), containing abundant carboxyl and hydroxyl groups, can typically inhibit degradation processes by occupying metal active sites or scavenging radicals [59]. However, as shown in Figure 11d, BPA degradation efficiency remained largely unaffected even in the presence of 1–5 mg L^−1^ HA. This contrasts with the pronounced inhibition often reported for transition-metal-based catalysts [54,60,61]. The observed inertness likely stems from the abundant surface −OH groups on Mg(OH)_2_, which prevent direct binding between highly deprotonated HA and Mg(II) sites, thereby preserving the catalytic activity [34]. Furthermore, the catalytic action proceeds via surface hydroxyl groups activating PMS to generate ^1^O_2_, a pathway for which HA presents minimal competition or scavenging. Collectively, these findings demonstrate that PMS activation via surface hydroxyl groups to generate ^1^O_2_ offers superior robustness against both organic and inorganic interferences compared to conventional transition-metal-driven radical pathways.

### 3.6. Reusability and Stability of Mg(OH)_2_

**Figure 12:**
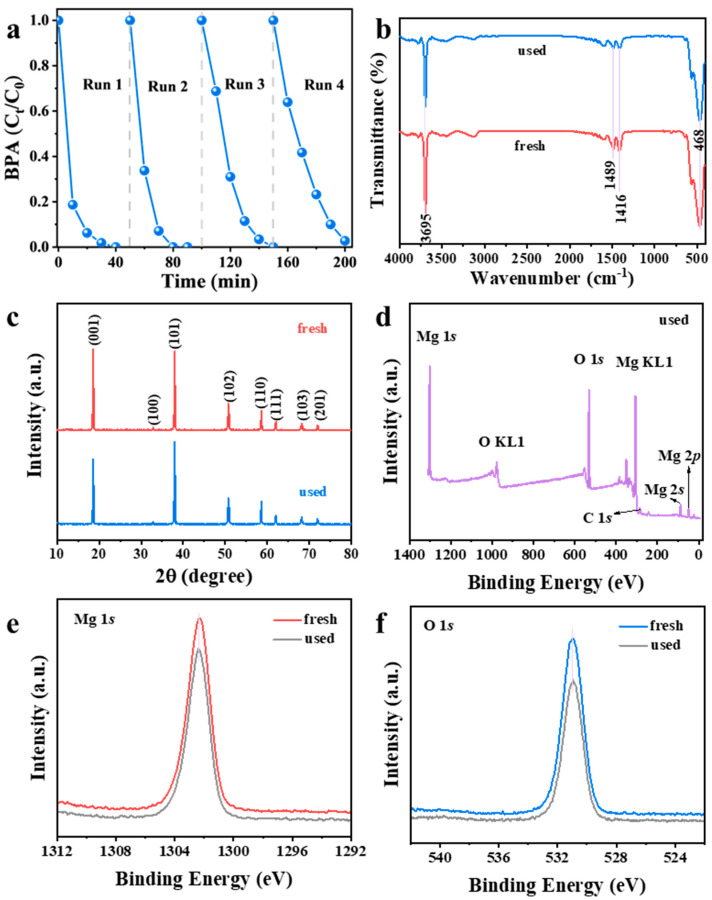
(**a**) Reuse stability of Mg(OH)_2_ for PMS activation in BPA degradation. (**b**) FT−IR spectra, (**c**) XRD patterns, and (**d**) XPS survey spectra of fresh and used Mg(OH)_2_. High-resolution XPS spectra of (**e**) Mg 1s and (**f**) O 1s for fresh and used Mg(OH)_2_.

The reusability of Mg(OH)_2_ was evaluated over four consecutive catalytic cycles. After each run, the spent catalyst was recovered via centrifugation, thoroughly washed with ethanol and deionized water, and dried under vacuum before reuse under identical conditions. As shown in Figure 12a, the catalyst maintained excellent performance, achieving a BPA degradation efficiency of 98% even in the fourth run. FTIR analysis (Figure 12b) confirmed the integrity of the catalyst’s chemical structure after reuse, with all characteristic peaks—including the O–H stretching vibration (~3698 cm^–1^), the bands in the 1400–1500 cm^–1^ region, and the broad Mg–O/O–H bending band (400–600 cm^–1^)—remaining unchanged. Notably, the absence of new absorption peaks indicates no formation of additional functional groups and, importantly, no persistent adsorption of reaction intermediates or products on the catalyst surface after washing. The structural and chemical stability was further confirmed by material characterization. The XRD pattern of the used catalyst (Figure 12c) remained identical to that of the fresh Mg(OH)_2_, confirming the preservation of its hexagonal brucite structure. XPS analysis provided additional evidence of stability. The XPS survey scan of the used Mg(OH)_2_ (Figure 12d) displayed predominant peaks for Mg, O, and C, with the latter assigned to adventitious carbon used for binding energy calibration. The high-resolution Mg 1s spectrum (Figure 12e) displayed a peak at 1302.4 eV, corresponding to Mg^2+^ in Mg(OH)_2_, while the O 1s spectrum (Figure 12f) showed a dominant peak at 531.0 eV, attributable to oxygen in hydroxyl groups (–OH). Both spectral features were unchanged compared to the pristine catalyst. These results collectively demonstrate the outstanding reusability and structural integrity of Mg(OH)_2_ in the PMS activation process.

## 4. Conclusions

In summary, this study demonstrates that Mg(OH)_2_ serves as an effective catalyst for PMS activation through a nonradical pathway predominantly involving ^1^O_2_ generation. The system achieves rapid and complete degradation of electron-rich pollutants such as BPA within 40 min while exhibiting exceptional pH self-regulation, stabilizing the solution at ~9.8 and maintaining high catalytic efficiency across a broad initial pH range (3–11). The Mg(OH)_2_ catalyst exhibits outstanding performance in PMS activation, primarily driven by its surface hydroxyl groups. The system presents several notable practical advantages: (1) inherent pH-buffering capacity that ensures robust catalytic activity over a wide pH range, overcoming a key limitation in conventional PMS-based processes; (2) substrate-selective oxidation, enabling rapid degradation of electron-rich contaminants while demonstrating resilience toward electron-poor compounds and common matrix interferents (e.g., inorganic anions and natural organic matter); (3) integrated flocculation functionality, facilitating the aggregation and removal of oxidation intermediates; and (4) excellent structural and chemical stability over repeated cycles, highlighting its reusability. Beyond elucidating a surface hydroxyl-mediated nonradical activation mechanism, this work establishes Mg(OH)_2_ as a low-cost, stable, and multifunctional material. The findings provide a novel strategy for designing advanced oxidation systems that simultaneously enhance catalytic efficiency, operational stability, and post-treatment management, offering significant potential for sustainable wastewater treatment applications.

## Data Availability

The original contributions presented in this study are included in the article/Supplementary Material. Further inquiries can be directed to the corresponding author.

## Supplementary Materials

The following supporting information can be downloaded at: https://www.mdpi.com/article/10.3390/nano16030166/s1, Table S1: Comparison of catalytic performance of Mg(OH)_2_ with other catalysts on PMS activation for BPA degradation by ^1^O_2_ process. References [62,63,64,65,66,67,68,69,70,71,72] are cited in the supplementary materials.
