# Supplementary material for: pH-Self-Buffering and Flocculation-Enabled Nonradical Oxidation via Magnesium Hydroxide-Activated Peroxymonosulfate for Selective Organic Pollutant Degradation
Source: Nanomaterials (Basel). 2026 Jan 26;16(3):166. doi: 10.3390/nano16030166 (PMC12899425; doi:10.3390/nano16030166)
Supplement: Supplementary file 1 [file nanomaterials-16-00166-s001.zip › nanomaterials-4097444-supplementary.pdf]

Supporting Information for

pH-Self-Buffering and Flocculation-Enabled Nonradical Oxidation via Magnesium Hydroxide-Activated Peroxymonosulfate for Selective Organic Pollutant Degradation

Yunfeng Zhang[1,3,4], Cheng Zhao[1,3,4], Zhongqun Li[2], Dexin Kong[1,3,4], Lingshuai Kong[1,2*]

[1]Shandong Provincial Geo-mineral Engineering Exploration Institute, Shandong Provincial Bureau of Geology & Mineral Resources, Jinan, 250014 China

[2]Institute of Eco-Environmental Forensics, School of Environmental Science and Engineering, Shandong University, Qingdao, 266237 China

[3]Shandong Engineering Research Center for Environmental Protection and Remediation on Groundwater, Jinan, 250014 China

[4]Key Laboratory of Geological Disaster Risk Prevention and Control，Emergency Management Department of Shandong Province, Jinan, 250014 China

* Corresponding author: E-mail: qfkongls@sdu.edu.cn

Table S1 Comparison of catalytic performance of $Mg(OH)_2$ with other catalysts on PMS activation for BPA degradation by $^1O_2$ process.

Catalysts	Experimental conditions	Degradation time (efficiency)	Ref.
Natural bamboo-derived porous carbon	$[BPA]_0$ = 10 mg L^{-1}, [Catalyst] = 0.9 mg L^{-1}, [PMS] = 0.9 mM	96.5% (100 min)	[1]
Fe^0-montmorillonite	[BPA] = 25 mg L^{-1}, [Catalyst] = 0.4 g L^{-1}, [PMS] = 1 mM	99.3% (120 min)	[2]
Periclase	[BPA] = 5 mg L^{-1}, [Catalyst] = 0.5 g L^{-1}, [PMS] = 0.6 mM	100% (40 min)	[3]
SA-Mn-NC	[BPA] =20 mg L^{-1}, [Catalyst] = 50 mg L^{-1}, [PMS] = 0.4 mM	95% (2 min)	[4]
BiOBr	[BPA] = 20 mg L^{-1}, [Catalyst] = 0.5 g L^{-1}, [PDS] = 1 mM	90% (15 min)	[5]
Hierarchical nano-vesicles with bimetal-encapsulated	[BPA] = 20 mg L^{-1}, [Catalyst] = 0.5 g L^{-1}, [PMS] = 1 mM	100% (15 min)	[6]
CNT-FePc Nanohybrid Filter	[BPA] = 10 mg L^{-1}, flow rate = 2.0 mL L^{-1}, [PMS] = 2 mM	98.4%	[7]

M4F1-MnOx	[BPA] = 5 mg L^{-1}, [Catalyst] = 0.2 g L^{-1}, [PMS] = 0.2 mM	64% (20 min)	[8]
layered crednerite	[BPA] = 10 mg L^{-1}, [Catalyst] = 0.1 g L^{-1}, [PMS] = 0.6 mM	100% (50 min)	[9]
2D Cobalt-carbon–nitrogen loaded 3D Prussian blue analogues	[BPA] = 20 mg L^{-1}, [Catalyst] = 0.15 g L^{-1}, [PMS] = 1 mM	90% (60 min)	[10]
graphite phase carbon nitride	[4-CP] = 5 mg L^{-1}, [Catalyst] =1 g L^{-1}, [PMS] = 4 mM	100% (80 min)	[11]
carbon nitride/biochar composite	[BPA] = 8 mg L^{-1}, [Catalyst] =1 g L^{-1}, [PMS] = 1 mM	96.6% (60 min)	[12]
$Mg(OH)_2$	[BPA] = 5 mg L^{-1}, [Catalyst] = 0.5 g L^{-1}, [PMS] = 0.6 mM	100% (40 min)	This work

References

1. Zhao, L.; Jin, C.; Liu, H.; Yang, Z.; Liu, Y. Natural bamboo-derived porous carbon activates PMS for BPA degradation: A singlet oxygen-dominated nonradical pathway. *Journal of Environmental Sciences* **2026**.
2. Yang, S.; Wu, P.; Liu, J.; Chen, M.; Ahmed, Z.; Zhu, N. Efficient removal of bisphenol A by superoxide radical and singlet oxygen generated from peroxymonosulfate activated with Fe0-montmorillonite. *Chemical Engineering Journal* **2018**, *350*, 484-495.
3. Kong, L.; Fang, G.; Xi, X.; Wen, Y.; Chen, Y.; Xie, M.; Zhu, F.; Zhou, D.; Zhan, J. A novel peroxymonosulfate activation process by periclase for efficient singlet oxygen-mediated degradation of organic pollutants. *Chemical Engineering Journal* **2021**, *403*, 126445.
4. Jia, Y.; Chen, Y.; Xue, Y.; Fan, J. Efficient activation of peroxymonosulfate by Mn single-atom: Critical role of Mn-N4 coordination for generating singlet oxygen. *Separation and Purification Technology* **2024**, *335*, 126129.
5. Bu, Y.; Li, H.; Yu, W.; Pan, Y.; Li, L.; Wang, Y.; Pu, L.; Ding, J.; Gao, G.; Pan, B. Peroxydisulfate Activation and Singlet Oxygen Generation by Oxygen Vacancy for Degradation of Contaminants. *Environmental Science & Technology* **2021**, *55*, 2110-2120.
6. Lyu, Z.; Xu, M.; Wang, J.; Li, A.; François-Xavier Corvini, P. Hierarchical nano-vesicles with bimetal-encapsulated for peroxymonosulfate activation: Singlet

oxygen-dominated oxidation process. *Chemical Engineering Journal* **2022**, *433*, 133581.

7. Xie, C.; Ren, Y.; Liu, Y. Singlet Oxygen-Mediated Micropollutant Degradation Using an FePc-Modified CNT Filter via Peroxymonosulfate Activation. *Catalysts* **2025**, *15*, 747.

8. Zeng, H.; Yang, B.; Zhang, J.; Zhu, H.; Deng, J.; Shi, Z.; Zhou, S.; Zhang, H.; Cai, A.; Deng, L. MnFe layered double hydroxides confined MnOx for peroxymonosulfate activation: A novel manner for the selective production of singlet oxygen. *Environmental Pollution* **2024**, *348*, 123865.

9. Sui, C.; Nie, Z.; Liu, H.; Boczkaj, G.; Liu, W.; Kong, L.; Zhan, J. Singlet oxygen-dominated peroxymonosulfate activation by layered crednerite for organic pollutants degradation in high salinity wastewater. *Journal of Environmental Sciences* **2024**, *135*, 86-96.

10. Chen, Y.; Chen, P.; Qin, Z.; Jing, C.; Zhu, Y.; Liu, R. 2D Cobalt-carbon–nitrogen loaded 3D Prussian blue analogues enhances peroxymonosulfate activation for bisphenol A Degradation: Singlet oxygen dominated nonradical pathway. *Separation and Purification Technology* **2024**, *347*, 127636.

11. Zhang, X.; Zhan, J.; Ma, J.; Wang, Z.; Han, B.; Li, F.; Zhang, Y.; Yang, Z. Metal-free catalysts with local fluorination regulation for peroxymonosulfate activation: Nearly 100 % singlet oxygen production for selective degradation of aqueous organic pollutants. *Chemical Engineering Journal* **2024**, *480*, 148026.

12. Wang, H.; Guo, W.; Si, Q.; Liu, B.; Zhao, Q.; Luo, H.; Ren, N. Non-covalent doping of carbon nitride with biochar: Boosted peroxymonosulfate activation performance and unexpected singlet oxygen evolution mechanism. *Chemical Engineering Journal* **2021**, *418*, 129504.
